# Interannual characteristics and driving mechanism of CO_2_ fluxes during the growing season in an alpine wetland ecosystem at the southern foot of the Qilian Mountains

**DOI:** 10.3389/fpls.2022.1013812

**Published:** 2022-10-19

**Authors:** Jingbin Zhu, Hongqin Li, Huidan He, Fawei Zhang, Yongsheng Yang, Yingnian Li

**Affiliations:** ^1^ College of Tourism, Resources and Environment, Zaozhuang University, Zaozhuang, China; ^2^ Key Laboratory of Adaptation and Evolution of Plateau Biota, Northwest Institute of Plateau Biology, Chinese Academy of Sciences, Xining, China; ^3^ College of Life Sciences, Luoyang Normal University, Luoyang, China

**Keywords:** Alpine wetland, CO_2_ fluxes, growing season, driving mechanism, Qinghai–Tibetan plateau

## Abstract

The carbon process of the alpine ecosystem is complex and sensitive in the face of continuous global warming. However, the long-term dynamics of carbon budget and its driving mechanism of alpine ecosystem remain unclear. Using the eddy covariance (EC) technique—a fast and direct method of measuring carbon dioxide (CO_2_) fluxes, we analyzed the dynamics of CO_2_ fluxes and their driving mechanism in an alpine wetland in the northeastern Qinghai–Tibet Plateau (QTP) during the growing season (May–September) from 2004–2016. The results show that the monthly gross primary productivity (GPP) and ecosystem respiration (Re) showed a unimodal pattern, and the monthly net ecosystem CO_2_ exchange (NEE) showed a V-shaped trend. With the alpine wetland ecosystem being a carbon sink during the growing season, that is, a reservoir that absorbs more atmospheric carbon than it releases, the annual NEE, GPP, and Re reached −67.5 ± 10.2, 473.4 ± 19.1, and 405.9 ± 8.9 gCm^-2^, respectively. At the monthly scale, the classification and regression tree (CART) analysis revealed air temperature (Ta) to be the main determinant of variations in the monthly NEE and GPP. Soil temperature (Ts) largely determined the changes in the monthly Re. The linear regression analysis confirmed that thermal conditions (Ta, Ts) were crucial determinants of the dynamics of monthly CO_2_ fluxes during the growing season. At the interannual scale, the variations of CO_2_ fluxes were affected mainly by precipitation and thermal conditions. The annual GPP and Re were positively correlated with Ta and Ts, and were negatively correlated with precipitation. However, hydrothermal conditions (Ta, Ts, and precipitation) had no significant effect on annual NEE. Our results indicated that climate warming would be beneficial to the improvement of GPP and Re in the alpine wetland, while the increase of precipitation can weaken this effect.

## Introduction

Global climate change is predicted to have a substantial influence on the stability of the grassland ecosystem, an ecosystem whose vegetation is dominated by grasses (Piao et al., 2012; Chai et al., 2019; Chen et al., 2021a). The carbon process of the alpine grassland ecosystem is extremely sensitive to and complex in the face of continuous global warming (Shen et al., 2015; Wang et al., 2017). Because the soil of the alpine ecosystem contains a substantial amount of thatch, or undecomposed organic matter, it is particularly vulnerable to changes in the global climate (Shen et al., 2022). Although high-altitude grassland ecosystems have attracted more attention recently, studies on carbon processes based on long-term data have still been lacking (Li et al., 2021).

The unique environmental conditions in the alpine grassland, namely, its high altitude, low temperature, and strong radiation, aid in the carbon fixation of vegetation, whereby vegetation converts inorganic carbon into organic compounds (Shen et al., 2022). However, the differences between biotic and abiotic factors still significantly impacted carbon source/sink dynamics, which will increase the uncertainty of predicting the carbon balance dynamics of the alpine grassland under the background of climate change in the future (Chai et al., 2017). Studies have shown that increases in the temperature and CO_2_ concentration improve the photosynthetic production capacity of vegetation, referring to the rate at which vegetation can fix carbon during photosynthesis, in alpine grassland ecosystems (Shen et al., 2015; Kang et al., 2022). However, temperature increases also stimulate the decomposition of soil organic matter and enzymatic activities of microorganisms, thus promoting the release of soil carbon (Li et al., 2019). Furthermore, the temperature increase leads to an increase in soil water evaporation, which worsens drought stress on vegetation and can thus lead to a decrease in the amount of CO_2_ fixed by vegetation through photosynthesis (Baldocchi et al., 2021). The balance between these opposing biological and metabolic processes determines the feedback effect of the alpine ecosystem on the climate environment; thus, the influence of climate change on the source/sink dynamics of alpine ecosystems in the QTP is still unclear (Chen et al., 2021a).

Studies have shown that CO_2_ fluxes in alpine ecosystems are mainly controlled by the dynamics of carbon balance in the growing season (Chai et al., 2017; Chai et al., 2019). In the context of global climate change, the rise of temperature is expected to promote snow melt and vegetation greening earlier, thus prolonging the length of the growing season (Groendahl et al., 2007; Street et al., 2007; Shen et al., 2022). Wetland ecosystems are fragile and play an important role in the carbon cycle of global terrestrial ecosystems (Temmink et al., 2022; Zhang et al., 2022). The wetland in QTP occupies 33% of China’s wetland area (Zheng et al., 2013; Kang et al., 2022). Furthermore, permafrost thawing and glacier retreat may create larger or new wetlands on the QTP (Kang et al., 2014; Shen et al., 2022). Climatic and environmental factors at different time scales may have different impacts on the carbon cycle (Saito et al., 2009; Zhu et al., 2015; Zhu et al., 2020). However, previous studies on the carbon cycle of alpine ecosystems have been conducted over short periods; hence, how the carbon balance in the alpine wetland ecosystem responds over the long term to climate change remains unclear (Li et al., 2021). Hence, in this work, we analyze continuous data of 13 years, measured by the eddy covariance (EC) technique in an alpine wetland of the northeastern QTP. This study aimed to measure the interannual variation of CO_2_ fluxes in alpine wetland ecosystems and clarify the driving mechanisms of major environmental factors so as to provide a theoretical basis for predicting the carbon budget of alpine wetland ecosystems amidst climate change in the future.

## Materials and methods

### Study area

The study site was located at the southern foot of the Qilian Mountains (37°35′N, 101°20′E, 3250 m a.s.l.). The region mainly has a plateau continental monsoon climate. Previous monitoring data found the annual average temperature to be −1.1°C, and the average minimum (−18.3°C) and maximum (12.6°C) temperature were recorded in January and July, respectively. The mean annual precipitation was approximately 490 mm, and precipitation in the growing period accounted for over 80% of the total annual precipitation. The study area soil is a silty clay loam of Mat-Cryic Cambisols, which is rich in organic matter. *Carex pamirensis* is the alpine wetland’s constructive species. Many mounds are scattered across the study site, owing to the seasonal freeze–thaw process (Song et al., 2015; Wang et al., 2017).

### Flux and abiotic measurements

The EC sensor array comprised a three-dimensional (3D) ultrasound anemometer (CSAT3, Campbell, Scientific Inc., Logan, UT, USA) and an open-path infrared CO_2_/H_2_O analyzer (Li7500, Li-cor Inc., Lincoln, Nebraska, USA). The raw data is recorded by the data collector at a frequency of 10 Hz. A data logger (CR5000, Campbell Scientific Inc.) was used to calculate and log the mean, variance, and covariance values of the raw data every 30 min. The growing season is from May to September (Zhang et al., 2008).

### Data processing

We applied the flux data processing method from ChinaFLUX (Yu et al., 2008; Fu et al., 2009). We obtained the daily GPP by subtracting the net ecosystem CO_2_ exchange (NEE) from the ecosystem respiration (Re) (Equation (1)); daily Re was the sum of nocturnal respiration (Re_n_) and daytime respiration (Re_d_), which was extrapolated from the exponential regressions of Re_n_ with nighttime soil temperature to the daytime periods (Yu et al., 2008).


(1) $$\text{GPP}=\text{Re}-\text{NEE}=({\text{Re}}_{\text{d}}+{\text{ Re}}_{\text{n}})-\text{NEE}$$

### Statistical analysis

The classification and regression tree (CART) is used to determine which environmental factors—such as air temperature (Ta), soil temperature in 5cm depth (Ts), photosynthetic photon flux density (PPFD), precipitation (PPT), air relative humidity (RH), and vapor pressure deficit (VPD)—function in a major controlling manner in variations in CO_2_ fluxes. We used SYSTAT 13.0 (Systat Software Inc, USA) for the CART and linear regression analyses.

## Results

### Variation characteristics of climatic factors

**Figure 1:**
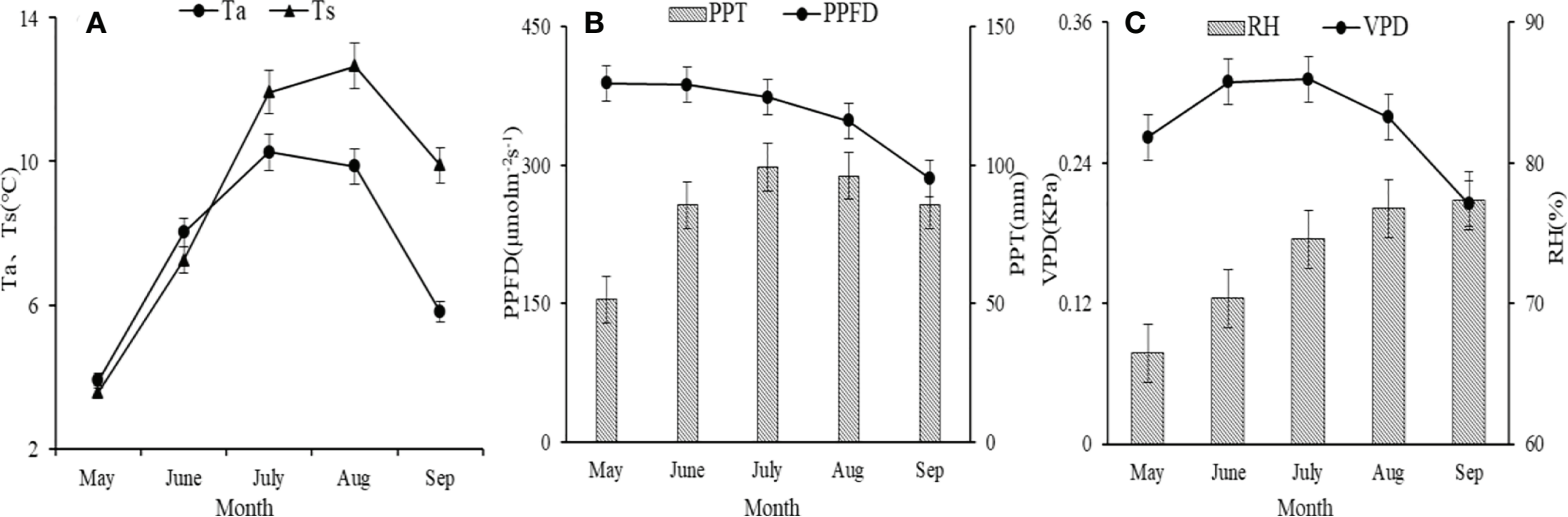
The average value of monthly air temperature (Ta) (± its standard deviation) and soil temperature (Ts) **(A)**; photosynthetic photon flux density (PPFD) and precipitation (PPT) **(B)**; vapor pressure deficit (VPD) and air relative humidity (RH) **(C)**.

The average monthly Ta, Ts, VPD, and PPT showed a unimodal trend, but the peak did not occur in consistent months (**Figure 1**). The peak values of monthly Ta, PPT, and VPD occurred in July, but the maximum value of monthly Ts occurred in August (**Figure 1**). Only at the beginning of the growing season (May, June), monthly Ts was higher than Ta (**Figure 1A**). The mean values of annual Ta, Ts, and PPT in the growing season were 7.5 ± 1.0, 9.5 ± 1.2°C, and 418.2 ± 34.0 mm, respectively. Monthly PPFD and RH showed the opposite trend: Monthly PPFD gradually decreased, and monthly RH gradually increased in the growing season (**Figures 1B, C**).

### Variation characteristics of CO_2_ fluxes

**Figure 2:**
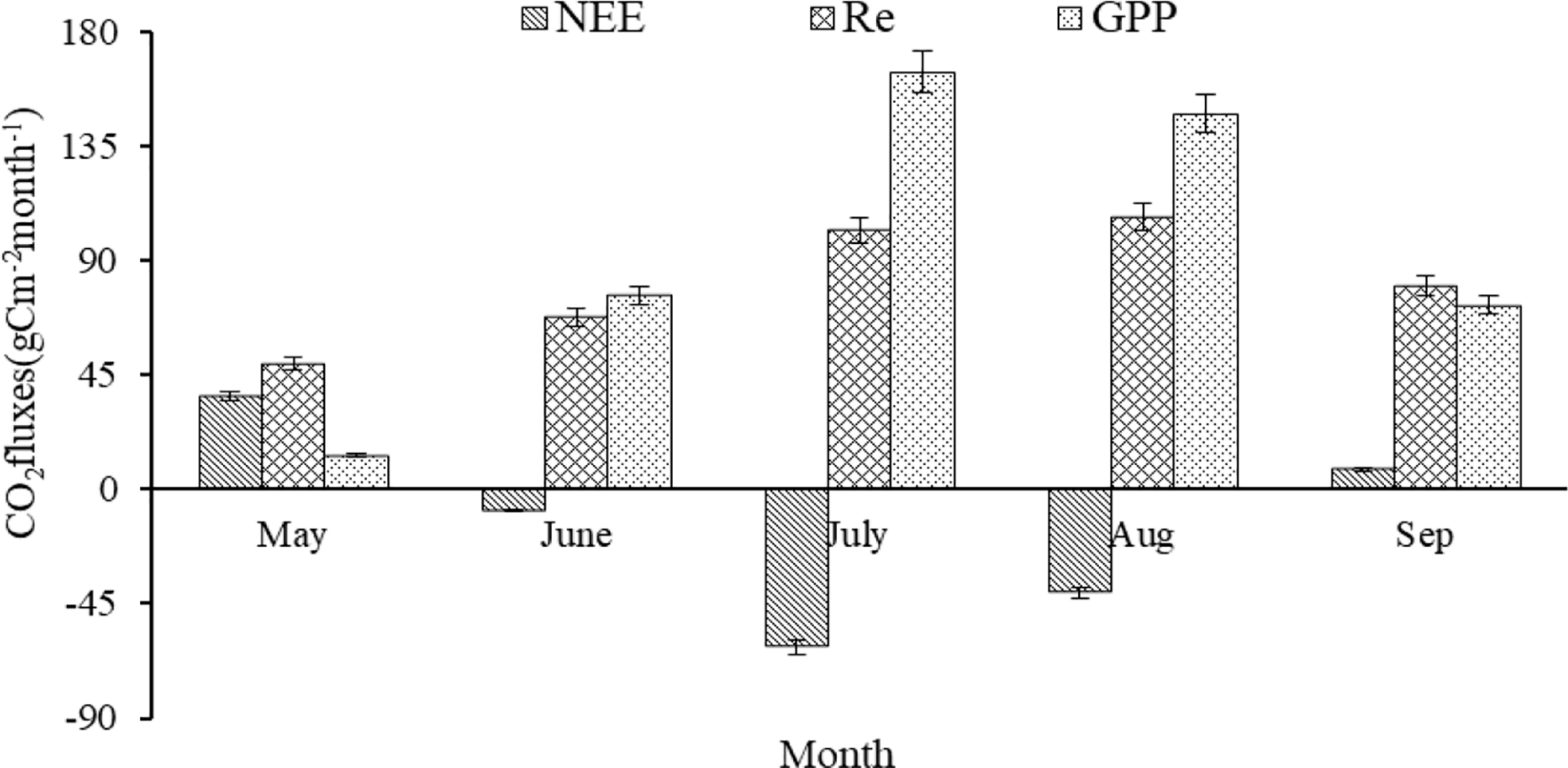
The average value of monthly CO_2_ fluxes (gCm^-2^month^-1^) of alpine wetland in the growing season for the period 2004-2016.

**Figure 3:**
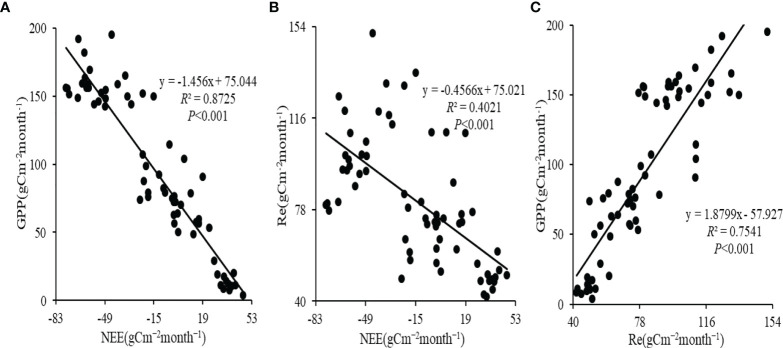
Linear regressions between monthly GPP and NEE **(A)**, linear regressions between monthly Re and NEE **(B)**, and linear regressions between monthly GPP and Re **(C)** of alpine wetland in the growing season for the period 2004-2016.

The average monthly GPP and Re showed a unimodal trend (**Figure 2**). The peak of the monthly GPP (164.2 ± 15.1gCm^−2^month^−1^) occurred in July, but the maximum of the monthly Re (107.0 ± 16.9 gCm^−2^month^−1^) occurred in August. The monthly NEE showed a V-shaped trend, and the minimum of the monthly NEE (-62.2 ± 9.5gCm^−2^month^−1^) appeared in July. Only in May of the growing season, when the NEE was positive, did the alpine wetland act as a carbon source (**Figure 2**). In addition, the monthly NEE was negatively correlated with both monthly GPP and monthly Re (*P*<0.001) (**Figures 3A, B**), while the monthly GPP was positively correlated with monthly Re (*P*<0.001) (**Figure 3C**). Compared with monthly Re (*R* ^2^ = 0.40), the monthly GPP (*R* ^2^ = 0.87) has a stronger control effect on the monthly NEE during the growing season (**Figures 3A, B**).

**Figure 4:**
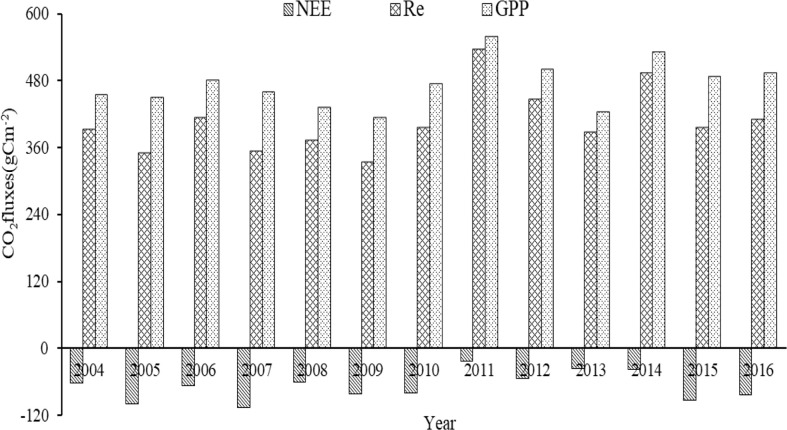
The annual CO_2_ fluxes of alpine wetland in the growing season for the period 2004-2016.

The mean values of annual GPP and Re in the growing season of alpine wetland from 2004 to 2016 were 473.4 ± 19.1 gCm^−2^ and 405.9 ± 8.9 gCm^−2^, respectively (**Figure 4**). The mean annual NEE of the alpine wetland in the growing season was −67.5 ± 10.2 gCm^−2^ (**Figure 4**), which represented a carbon sink, with the maximum and minimum values of −22.6 gCm^−2^ (2011) and −105.4 gCm^−2^ (2007), respectively. Linear regression analysis showed that annual GPP was positively correlated with Re (*P*<0.001), and annual NEE was positively correlated with Re (*P*=0.003), but NEE was not significantly correlated with GPP (*P*=0.158). Therefore, annual Re might be more important than GPP for the dominant role of NEE at the interannual scale.

### Effects of climatic factors on CO_2_ fluxes at the monthly scale

**Figure 5:**
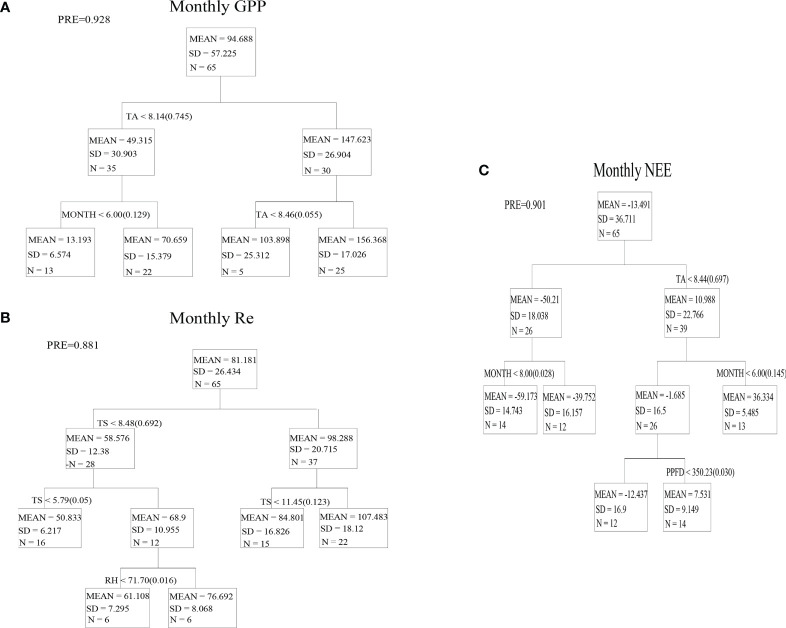
Regression trees for monthly GPP **(A)**, Re **(B)** and NEE **(C)** from environmental variables of alpine wetland in the growing season (May-September).

The CART analysis results reveal that Ta and Ts were the main controlling factors of the monthly GPP and Re, respectively (**Figures 5A, B**). Ta could explain 92.8% of the variation in the monthly GPP (**Figure 5A**), and Ts could explain 88.1% of the variation in the monthly Re (**Figure 5B**). The results of CART reveal that Ta was the dominant factor of monthly NEE, and Ta could explain 90.1% of the variation in the monthly NEE (**Figure 5C**). Linear regression analysis also showed that thermal conditions (Ta, Ts) were the main controlling factors of monthly CO_2_ fluxes (**Table 1**). In sum, Ta had a major impact on the change in the monthly GPP and NEE, and Ts was the main controlling factor of Re. This also reveals that GPP rather than Re was the predominant determinant of the change in NEE at the monthly scale.

**Table 1:** Linear regressions between monthly CO_2_ fluxes and environmental variables of alpine wetland in the growing season (May-September).

Month	GPP			Re			NEE		
	Linear Equation	*R* ^2^	*P*	Linear Equation	*R* ^2^	*P*	Linear Equation	*R* ^2^	*P*
Ta(x_1_)	y = 19.86x_1_-54.55	0.80	0.000	y = 7.27x_1_ + 26.53	0.50	0.000	y = -12.60x_1_+81.16	0.78	0.000
RH(x_2_)	y = 7.14x_2_-427.1	0.37	0.000	y = 3.34x_2_ – 163.12	0.38	0.000	y = -3.81x_2_+264.54	0.26	0.000
PPFD(x_3_)	y = -0.15x_3_+149.06	0.02	0.313	y = -0.17x_3_ + 142.93	0.10	0.011	y = -0.02x_3_-6.27	0.00	0.835
Ts(x_4_)	y = 12.69x_4_-19.76	0.70	0.000	y = 5.93x_4_ + 27.69	0.72	0.000	y = -6.77x_4_+47.53	0.48	0.000
PPT(x_5_)	y = 1.25x_5_-9.72	0.35	0.000	y = 0.48x_5_+41.25	0.24	0.000	y = -0.77x_5_+51.03	0.32	0.000
VPD(x_6_)	y = 403.57x_6_-15.77	0.12	0.005	y = 89.52x_6_+56.68	0.03	0.189	y = -313.84x_6_+72.40	0.17	0.001

### Effects of climatic factors on CO_2_ fluxes at the interannual scale

**Figure 6:**
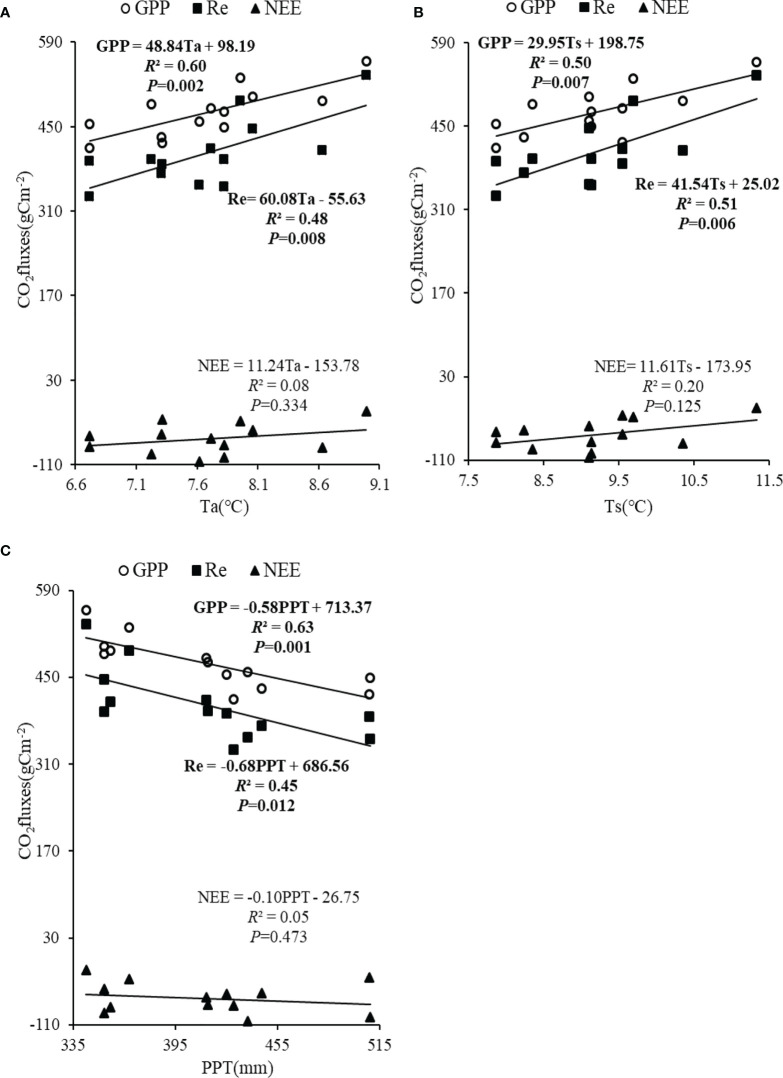
Linear regressions between seasonal CO_2_ fluxes and Ta **(A)**, linear regressions between seasonal CO_2_ fluxes and Ts **(B)**, and linear regressions between seasonal CO_2_ fluxes and PPT **(C)**.

At the interannual scale, linear regression analysis showed that VPD, RH, and PPFD had no significant correlation with annual CO_2_ fluxes during the growing season (*P*>0.05). The annual GPP and Re were positively correlated with Ta and Ts (*P*<0.01) (**Figures 6A, B**). However, Ta and Ts had no significant correlation with the annual NEE (*P*>0.05) (**Figures 6A, B**). The PPT was negatively correlated with the annual GPP and Re (*P*<0.05), but had no significant correlation with the annual NEE (*P*>0.05) (**Figure 6C**).

## Discussion

### Driving mechanisms of CO_2_ fluxes at the monthly scale

CART and linear regression analysis showed that Ta in the growing season of alpine wetland was the most important controlling factor for the monthly GPP, which might be attributed to the relatively high aboveground biomass of alpine grassland vegetation. Thus, the growing season thermal conditions strongly impacted the photosynthesis of vegetation (Ueyama et al., 2013; Shen et al., 2015). Moreover, the thermal condition is the primary limiting factor to breaking the dormancy of vegetation and is crucial to the phenological development and sustainable metabolic growth of vegetation (Ueyama et al., 2013; Kang et al., 2022). Further, the growth and metabolism of vegetation in the alpine wetland ecosystem have full phenotypic plasticity to thermal conditions (Zhao et al., 2006; Li et al., 2021). In addition, soil microbial and enzyme activities in alpine grassland are extremely sensitive to temperature, and thermal conditions can indirectly affect the nutrient supply of soil for vegetation growth and metabolism by affecting microbial activities and enzyme activities (Li et al., 2021; Shen et al., 2022). The study of alpine meadows, alpine shrubs and alpine wetlands near the study site showed that the daily GPP during the growing season was mainly controlled by Ta and PPFD. However, when PPFD is relatively high and exceeds a certain threshold, GPP is almost unaffected by the increase of PPFD (Zhao et al., 2005). Under the same PPFD condition, the GPP of the three vegetation types from high to low were alpine meadows, alpine shrubs, and alpine wetlands (Zhao et al., 2006; Li et al., 2016). However, studies have shown that climate and environmental factors may have different effects on the photosynthetic production capacity of vegetation at different time scales (Zhu et al., 2020). Because PPFD reaches its peak in June and then begins to decline, there is no significant correlation between monthly PPFD and monthly GPP (*R* ^2^ = 0.02, *P*=0.313) during the growing season.

CART showed that the variation of monthly Re in alpine wetland was mainly controlled by Ts. Many studies have shown that soil temperature in alpine grassland ecosystems significantly affects CO_2_ release and nitrogen mineralization, and soil microbial biomass in alpine ecosystems is limited by low temperature (Song et al., 2015; Cao et al., 2019). The high soil temperature can stimulate microbial activities and enzyme activities and promotes soil respiration (Gao et al., 2011). Therefore, soil temperature becomes the primary controlling factor of CO_2_ emission from the alpine grassland ecosystem (Kang et al., 2022). However, some research has found that the increase in temperature improves autotrophic respiration, the loss of fixed carbon by plants, and inhibits heterotrophic respiration, the loss of fixed carbon by non-plant species, resulting in the poor response of Re to warming (De et al., 2008; Duan et al., 2019). CART and the linear regression analysis showed that Ts had a stronger controlling effect on the monthly Re in the alpine wetland than Ta, suggesting that soil respiration in the alpine wetland, compared to vegetation respiration, may be more sensitive to thermal conditions (**Figure 5** and **Table 1**).

Only in May of the growing season did the alpine wetland acts as a carbon source (**Figure 2**). Because the temperature is low and the precipitation is less in the early growing season of the alpine wetland, the vegetation just germinates and grows, and the growth metabolism of vegetation is weak (Zhao et al., 2010). Compared with ecosystem respiration, the photosynthetic production capacity of vegetation is low, so the alpine wetland is a carbon source in May. Linear regression analysis showed that monthly NEE was significantly negatively correlated with the monthly GPP and monthly Re during the growing season of alpine wetland from 2004 to 2016 (*P*<0.001) (**Figures 3A, B**), and compared with the monthly Re (*R* ^2^ = 0.40), the monthly GPP (*R* ^2^ = 0.87) had a stronger controlling effect on the monthly NEE. CART and linear regression analysis showed that the thermal conditions (Ta and Ts) of the alpine wetland were the dominant factors for the change in the monthly NEE at the monthly scale, and Ta had a stronger controlling effect on the monthly NEE. This is similar to the results of previous studies (Ueyama et al., 2013; Li et al., 2016; Chen et al., 2021a), suggesting that the carbon sequestration of alpine wetlands in the growing season is more dependent on the photosynthetic production capacity of vegetation at the monthly scale.

### Driving mechanisms of CO_2_ fluxes at the interannual scale

The mean annual NEE of the alpine wetland in the growing season was −67.5 ± 10.2 gCm^−2^ (**Figure 4**), which represented a carbon sink. Owing to the special climate of the QTP and the favorable water and thermal conditions in the growing season, the grassland plants have high primary production capacity (Kato et al., 2006; Luo et al., 2015). Moreover, owing to the relatively low temperature, especially the low temperature at night, vegetation respiration and soil respiration consume relatively less organic matter (Groendahl et al., 2007; Chai et al., 2017). Linear regression analysis showed that annual Re was more responsible than the GPP for the dominant role of NEE at the interannual scale, which indicates that the dominant factors of NEE are not consistent in different time scales. This may because there is a large amount of thatch in the soil of the alpine wetland, and the process of the microbial decomposition is sensitive to Ts, resulting in a stronger controlling effect of Re on NEE. This result indicates that soil respiration in the alpine wetland is crucial to carbon balance (Zhang et al., 2008; Zhao et al., 2010; Li et al., 2021).

Linear regression analysis showed that the annual GPP and Re were positively correlated with Ta and Ts (*P*<0.01) (**Figure 6A, B**). Temperature promoted photosynthetic productivity and autotrophic respiration of vegetation (Ueyama et al., 2013; Chen et al., 2021a). However, soil temperature promoted the decomposition of a large amount of organic matter in the soil and enhanced soil respiration (Zhao et al., 2010). In addition, the decomposition of soil organic matter provides nutrients for vegetation growth, which further strengthens the process of vegetation growth and metabolism (Zhang et al., 2008). The PPT was negatively correlated with the annual GPP, Re (*P*<0.05) (**Figure 6C**). The PPT could affect the depth of surface water in the alpine wetland, and the increase of PPT deepens the water depth to a certain extent (Wang et al., 2017). The surface water level of the wetland limits the movement of atmospheric oxygen into soil; thus, the microorganism activity is inhibited, the decomposition rate of soil organic matter is reduced, and soil respiration is reduced (Hirota et al., 2006). Furthermore, the photosynthesis of alpine wetland vegetation is reduced, owing to the reduction of nutrients in the soil (Chimner and Cooper 2003). Furthermore, the saturated zone of soil could also affect the soil heat transfer, thus impacting the change in soil temperature (Zhang et al., 2008; Zhang et al., 2022). Because soil temperature affects the decomposition rate of soil organic matter, water depth may indirectly affect GPP and Re by regulating soil temperatures (Zhao et al., 2010; Song et al., 2015; Chen et al., 2021b). However, owing to the similar responses of GPP and Re to hydrothermal conditions in the alpine wetland, there was no significant correlation between the NEE and hydrothermal conditions, indicating that it is necessary to be more cautious when evaluating the carbon source and sink capacity of alpine wetland. More in-depth studies are needed to verify this result (Niu et al., 2013; Peng et al., 2014; Li et al., 2021).

## Conclusions

Based on CO_2_ fluxes measured with the EC technique, the alpine wetland ecosystem was found to be a carbon sink during the growing season in the northeastern QTP. At the monthly scale, Ta and Ts played a crucial role in the dynamics of monthly CO_2_ fluxes. At the interannual scale, hydrothermal (Ta, Ts and PPT) conditions had significant effects on the GPP and Re, but had no significant effects on the NEE. Our results indicate that climate warming is beneficial to the improvement of GPP and Re during the growing season in the alpine wetland, while the increase of PPT may weaken this effect.

## Data availability statement

The raw data supporting the conclusions of this article will be made available by the authors, without undue reservation.

## Author contributions

JZ performed the research, analyzed data, and wrote the paper. HL, YY, and YL analyzed data and wrote the paper. HH and FZ conceived of the study. All authors have revised, discussed, and approved the final manuscript.

## Funding

This study was supported by Natural Science Foundation of Shandong Province (ZR2021QC222), the National Natural Science Foundation in China (32001185, 41877547), the Chinese Academy of Sciences-People’s Government of Qinghai Province Joint Grant on Sanjiangyuan National Park Research (YHZX-2020-07), the National Key R&D Program (2017YFA0604802), and the Qingtan Talent Scholar project in Zaozhuang University.

## Acknowledgments

The authors are grateful to Jinlong Wa for the help in obtaining field data.

## Conflict of interest

The authors declare that the research was conducted in the absence of any commercial or financial relationships that could be construed as a potential conflict of interest.

## Publisher’s note

All claims expressed in this article are solely those of the authors and do not necessarily represent those of their affiliated organizations, or those of the publisher, the editors and the reviewers. Any product that may be evaluated in this article, or claim that may be made by its manufacturer, is not guaranteed or endorsed by the publisher.
